# Protective effects of berberine in a rat model of polycystic ovary syndrome mediated via the PI3K/AKT pathway

**DOI:** 10.1111/jog.14730

**Published:** 2021-03-11

**Authors:** Jia Yu, Caifei Ding, Zhoujia Hua, Xuejuan Jiang, Chenye Wang

**Affiliations:** ^1^ Department of Reproductive Medicine Zhejiang Provincial Integrated Chinese and Western Medicine Hospital HangZhou City China

**Keywords:** berberine, insulin resistance, PI3K/AKT pathway, polycystic ovarian syndrome

## Abstract

**Background:**

Ber, a Chinese herbal monomer has been reported to exhibit an array of pharmacological activities related to the lowering of blood glucose and the treatment of polycystic ovarian syndrome (PCOS). In the present study, we aimed to elucidate the effect of berberine (Ber) on a rat model of PCOS mediated via the PI3K/AKT signaling pathway.

**Methods:**

A PCOS animal model was induced with the administration of letrozole, and animals were then randomized into untreated or Ber and metformin hydrochloride treated groups. After administration, fasting blood glucose, HOMA‐IR, fasting insulin (FINS) values, and the serum hormone levels were measured in PCOS rats. The ovarian tissues were stained with hematoxylin and eosin and terminal deoxynucleotidyl transferase‐mediated dUTP‐biotin nick end labeling (TUNEL) for pathological and apoptosis examination. Moreover, the effect of Ber on the proliferation and apoptosis of granulosa cells was detected by CCK‐8 assays and flow cytometry. The influence of Ber on granulosa cells was confirmed by blockade of the PI3K/AKT pathway. In addition, the modulatory effect of the blockade of the PI3K/AKT pathway on the expression of related proteins was demonstrated via western blotting.

**Results:**

We found that Ber was able to restore the serum hormone levels and improve IR in a PCOS rat model. The morphological lesions and apoptosis of the ovary were also restored by the Ber treatment. Blockade of the PI3K/AKT pathway attenuated the influences of Ber on the proliferation and apoptosis of granulosa cells.

**Conclusion:**

The beneficial effects of Ber on PCOS included alterations of the serum hormone levels, recovery of morphological lesions in the ovary, improvement of insulin resistance, and cell viability and inhibition of apoptosis, which were all mediated through the PI3K/AKT pathway.

## Background

Polycystic ovarian syndrome (PCOS) is an endocrine syndrome characterized by persistent anovulation, hyperandrogenemia, or insulin resistance (IR).^1^ It is the most common cause of infertility in women of childbearing age and affects approximately 4%–18% of all reproductive‐aged women around the world.^2^ At the same time, PCOS has also been defined as a metabolic syndrome, often associated with metabolic disorders like obesity and hyperlipidemia, and affected women are at a higher risk for type 2 diabetes, hypertension, and other cardiovascular diseases.^3^, ^4^ Clinically, the main causes of the reproductive and metabolic abnormalities in women with PCOS are hyperandrogenism and IR.^5^ However, the pathogenesis and mechanism of PCOS have not been fully elucidated.

Ber is an isoquinoline compound derived from many different plants like *Coptis chinensis Franch* and *Phellodendron chinensis var. glabriusculum*. It has been used in traditional Chinese medicine for many years and has been shown to be effective against IR and obesity, particularly against diabetes type 2 and hypercholesterolemia.^6^, ^7^ There is recent evidence indicating that Ber offers promise for treating PCOS‐associated IR. Several studies have indicated that Ber has similar effects as metformin in improving hyperglycemia and it is more beneficial for PCOS treatment.^8^ It has additional effects on body composition and hyperlipidemia in women with PCOS when compared with metformin.^9^

The PI3K/AKT signaling pathway is one of the most important insulin signaling pathways, which is not only closely related to IR, but also plays an important role in cell growth, proliferation, migration, invasion, inhibition of apoptosis, and angiogenesis.^10^, ^11^, ^12^ At present, several studies have indicated that Ber inhibits the mTOR pathway, which has abnormally high activity in the state of IR, mainly by activating the AMPK activity, to regulate the insulin signaling pathway and improve IR.^13^, ^14^ However, it remains uncertain whether Ber alters PCOS IR, promotes follicular cell proliferation, or has anti‐apoptotic effects by regulating the PI3K/AKT signaling pathway.

In the present study, a rat model of PCOS was constructed by daily gavage with a letrozole solution coupled with a high‐fat diet. Then, the ovarian granulosa cells of the model rats were extracted and cultured. The effects of Ber on IR, serum hormone levels, and ovary morphological lesions in the PCOS rats were explored, and the direct effects of Ber on the proliferation and apoptosis of granulosa cells were also investigated. In addition, LY294002 (a specific inhibitor of PI3K) and MK‐2206 (a specific inhibitor of AKT) were utilized to further verify the role of the PI3K/AKT signaling pathway on granulosa cell proliferation and apoptosis, along with an investigation of Ber's influence on the expression of related proteins.

## Methods

### Animals

Female Sprague–Dawley (SD) rats (21–23‐day‐old), were purchased from the Center of Experimental Animals at the Shanghai Sippr‐BK Laboratory Animal Co. Ltd. (Shanghai, China). All of the rats were housed at a standard room temperature of 23 ± 2°C and humidity of 55%–70% under a 12 h light/dark cycle with access to food and water. All animal procedures were conducted in accordance with the National Institutes of Health Guide for the Care and Use of Laboratory Animals. The animal experiments were performed according to the guidelines of laboratory animal care and were authorized by the Ethic Committee of Zhejiang Traditional Chinese Medicine University (Hangzhou, China). After the experiment, the rats were euthanized with carbon dioxide.

### In vivo study

#### PCOS model generation

After a week of adaptive feeding, 50 female SD rats were randomly assigned into five groups of 10 rats each: the control, PCOS, PCOS+ L‐Ber (95 mg/kg), PCOS+ H‐Ber (190 mg/kg), and metformin hydrochloride treated groups (PCOS+MH) (50 mg/kg).^15^ Due to the rat models prepared with letrozole exhibit similar endocrine and histological changes to those that occur in human PCOS, we used such a model to study the protective effects and mechanisms of Ber in PCOS rats. The PCOS, PCOS+H‐Ber, PCOS+L‐Ber, and PCOS+MH groups were administered by gavage of 1.0 mg/kg (0.4 mL) of letrozole dissolved in 1% carboxymethyl cellulose (CMC) solution once daily for 23 consecutive days. The control group was administered gavage of CMC solution. From 24 days, the PCOS+H‐Ber and PCOS+L‐Ber groups were administered by gavage of Ber, and the PCOS+MH group was administered by gavage of MH for four consecutive weeks. As a control, an equivalent volume of normal saline was injected subcutaneously into the rats in the control and PCOS groups. The control group rats were fed with conventional feed pellets while the PCOS, PCOS+ H‐Ber, PCOS+ L‐Ber, and PCOS+MH groups were fed with a high‐fat diet for 30 days. Starting at 31 days, vaginal smears were performed on each group, and their estrous cycles were observed for 15 consecutive days (about three sequential cycles). The fasting blood glucose (FBG), serum testosterone (T), insulin (INS) in rats were measured by tail vein blood collection after the model rats lost their estrous cycles. To evaluate the success of model‐building in the rats, we observed the complete estrous cycle and their serum T and INS.

#### Specimen collection

Twenty‐four hours after the last administration, blood from the rat aorta ventralis was centrifuged at 3000 rpm for 10 min, and following supernatant collection, the serum was stored at −80°C for subsequent use. A vaginal smear was conducted on 6‐week‐old rats, and the ovarian tissue was collected after fasting for a day. Pentobarbital sodium (1%, 50 mg/kg) was used for anesthesia, and then the bilateral ovaries were taken out. One ovary was rapidly fixed in 4% paraformaldehyde and the other was stored at −80°C until use.

#### Fasting blood glucose test and IR assay

To assess IR in the study animals, the rats were fasted for 12 h after the last administration, then FBG and FINS were assessed using venous blood samples by enzyme‐linked immunosorbent assay (ELISA). We followed the homeostasis model assessment of insulin resistance (HOMA‐IR) approach. HOMA‐IR was calculated using the following formula:

$$\text{HOMA}-\mathrm{IR}=\mathrm{FBG}\;\left(\text{mmol}/L\right)\times \text{FINS}\;\left(\mathrm{mU}/L\right)/22.5$$

#### Measurement of hormone level

We used ELISA kits (Enzyme Immunity Industry Co., Ltd.) to measure the serum concentrations of gonadotropins, including 17β‐estradiol (E2), follicle‐stimulating hormone (FSH), and luteinizing hormone (LH), and the steroid hormones, progesterone (P) and testosterone (T).

#### Rat ovarian morphology

At the study endpoint, the ovaries were quickly removed and dissected on dry ice, followed by formalin fixation/paraffin embedding; it was then cut into 4 μm sections and stained using hematoxylin and eosin (H&E). The morphological structure was observed under a light microscope and recognized by a morphological scoring standard^16^: 0 points: no injury; 1 point: inflammatory cell infiltration was less than 25%, the granular cell layer was more than 75%; 2 points: inflammatory cell infiltration ranged from 25% to 50%, the granular cell layer ranged from 50% to 75%; 3 points: inflammatory cell infiltration ranged from 50% to 75%, the granular cell layer ranged from 25% to 50%; 4 points: the inflammatory cell infiltration was more than 75%, the granular cell layer was less than 25%.

#### Ovarian tissue cell apoptosis

Part of ovarian tissue was fixed with 4% paraformaldehyde, routinely dehydrated, paraffin‐embedded in paraffin, cut into 4‐μm sections, dewaxed, cleared, and then subjected to TUNEL staining. Microscopically, the nuclei of the normal ovaries were blue, and the nuclei of the apoptotic cells were brown and yellow. The apoptotic index (AI) was expressed as the percentage of apoptotic cells in the total number of ovaries.

### In vitro study

#### Preparation of serum containing drugs

Twenty female SD rats were randomly assigned into 2 groups of 10 rats each: L‐Ber (95 mg/kg) and H‐Ber (190 mg/kg) group. These groups were administered gavage of 10 mL/kg of Ber once daily for seven consecutive days. One hour following the last intragastric administration, the abdominal artery blood was drawn from the rats in each group under aseptic conditions, placed at 4°C for 1 h, and centrifuged at 900 g and 4°C for 15 min. The drug‐containing serum samples from all of the rats in the same group were mixed, and then serum inactivation was conducted by placing it in a water bath which is 56°C for 30 min. Finally, the samples were sterilized by microporous membrane filtration, and then packed in tubes and stored at −70°C for the follow‐up experiments.

#### Isolation, culture, and identification of rat ovarian granulosa cells

The in vitro experiments were conducted according to the protocol described previously.^17^, ^18^ The successfully modeled rats were injected intraperitoneally (50 IU) with pregnant mare serum gonadotropin (PMSG, hor‐272, Prospec). The rats were fed routinely for 48 h and then killed. Under sterile conditions, the bilateral ovaries were removed with sterilized instruments, and the adipose tissue wrapped around the ovaries was removed. The ovaries were cleaned three times with sterile saline solution. Then, the ovaries were transferred to 3 mL serum‐free DMEM/F‐12 medium (HyClone), and the follicles were punctured with a 1‐mL syringe needle to release the granulosa cells. The granulosa cells were cultured in sterile bottles (2 × 10^6^ cells/bottle) using DMEM/F12 medium supplemented with 10% fetal bovine serum protein (FBS) at 37°C with 5% CO_2_. After 24 h of preculture, when the cells reached approximately 70% confluence, the medium was replaced with fresh media every 2 days. When the cells reached approximately 80% confluence, they were digested with 0.25% trypsin. The cells were fixed using 4% paraformaldehyde for 10 min; then, they were stained using H&E for the identification of the rat ovarian granulosa cells.

#### Cell counting kit‐8 viability assay

A cell counting kit‐8 (CCK‐8, MCE) was utilized to determine the granulosa cells viability and proliferation rates. Briefly, following exposure to various concentrations of Ber for various treatment times (0, 12 h, 24 h, 48 h, 72 h), 100 μL of cell suspension (approximately 5000 cells/well) was removed and 10 μL of CCK‐8 Solution was added to each well of a 96‐well plate. The plate was then incubated in a cell culture incubator. Finally, the optical density (OD value) was calculated with a microplate reader (CMaxPlus), and the absorbance of each experimental group was measured at 450 nm. Each treatment had three replicates. Cell viability = (A _Experiment_ − A _Blank_)/(A _Control_ − A _Blank_) × 100%.

#### Apoptosis analysis by flow cytometry

Apoptosis was evaluated using the Annexin V‐FITC Apoptosis Detection Kit (556 547, BD Pharmingen, China). Briefly, granulosa cells treated as above were collected and washed with ice‐cold PBS two times, and resuspended in 500 μL of binding buffer, then the mixture was centrifuged and the supernatant was discarded. The granulosa cells were resuspended in 100 μL Annexin V‐FITC binding solution, and stained for 15 min with 5 μL Annexin V‐FITC solution and 10 μL propidium iodide (PI) solution in the dark at room temperature. Finally, the apoptotic rate of the granulosa cells was measured by flow cytometry (FC500, Beckman Coulter, Inc.).

#### Western blotting analysis

The granulosa cells were homogenized in radioimmunoprecipitation assay (RIPA) lysis buffer (Beyotime), and centrifuged at 12 000*g* and 4°C for 5 min. The supernatant was collected, and the protein concentration in each group was detected using a bicinchoninic acid (BCA) protein quantification kit (pc0020). An appropriate amount of the protein sample was taken and mixed with 2 × sodium dodecyl sulfate (SDS) loading buffer (loading buffer: protein samples [v/v] = 4:1) to prepare the loading buffer system at an equal concentration, and the protein was denatured at 95°C for 5 min. Then, 10% sodium dodecyl sulfate‐polyacrylamide gel electrophoresis (SDS‐PAGE) gels were applied for electrophoresis under the constant voltage of 80 V. The protein was transferred onto a polyvinylidene difluoride (PVDF) membrane, which was blocked with freshly prepared 5% skim milk powder for 2 h and then washed. The membranes were then incubated with primary antibodies diluted according to the manufacturer's protocol overnight at 4°C, followed by incubation with a secondary antibody at room temperature for 1–2 h. The primary antibodies were directed against: GAPDH Antibody (1:1000, 60 004‐1‐1 g, Hangzhou Hua'an Biotechnology Co., Ltd.), Anti‐IGF1 Receptor Antibody (1:1000, ab182408, Abcam), Anti‐AKT (phospho T308) Antibody (1:1000, Ab32445, Abcam), Anti‐pan‐AKT Antibody (1:500, ab8805, Abcam), Anti‐Bad Antibody (1:2000, Ab32445, Abcam), Anti‐Bcl‐2 Antibody (1:2000, ab182858, Abcam), Bax Antibody (1:500, AF0120, Affinity), PI3 Kinase p85 Antibody (1:1000, 4292S, CST) and PI3 Kinase p110α Antibody (1:1000, 4255S, CST). The reactive bands were visualized with the ECL‐Plus reagent (Solarbio). The density of the band was quantified by Quantity One analytic software.

### Statistical analysis

The statistical analyses were performed using SPSS 16.0 (IBM). Values are expressed as $\bar{\chi }$ ± s. For the in vivo study, group comparisons were processed by using the LSD one‐way‐ANOVA test. For group comparisons in the in vitro study, we used Student's *t*‐test or the Kruskal–Wallis H test. In all cases, *p* < 0.05 was considered to indicate statistical significance.

## Results

### In vivo study

#### Berberine treatment altered HOMA‐IR and insulin sensitivity index values in PCOS model rats

**Figure 1:**
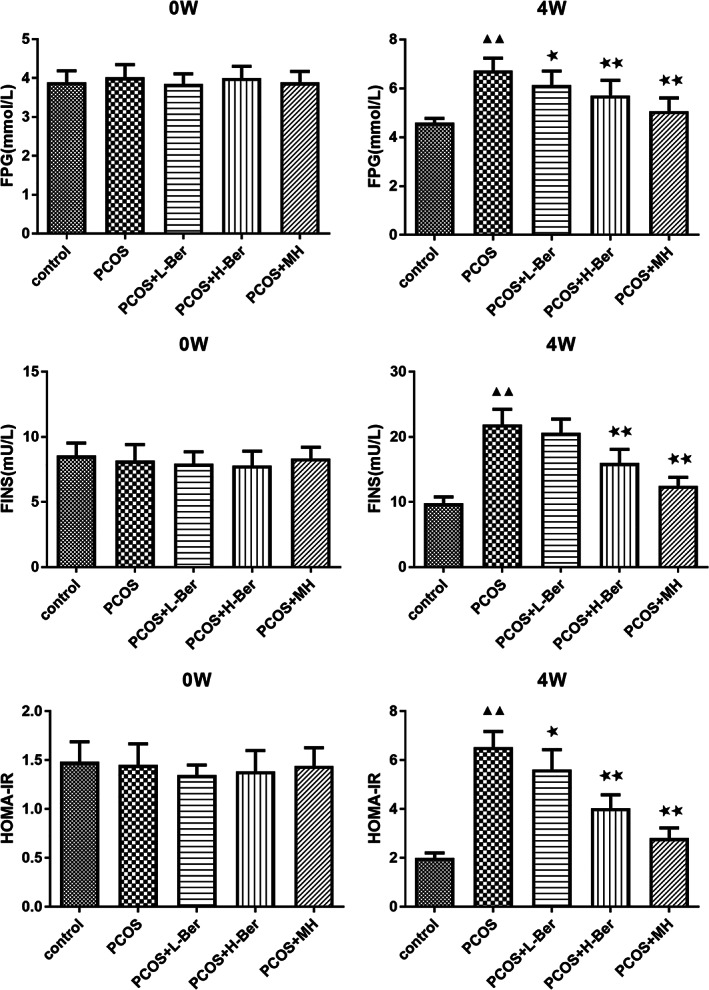
Effects of Ber and MH on the level of FPG, FINS, and HOMA‐IR in PCOS rats. (a), The levels of FPG, FINS, and HOMA‐IR in PCOS rats before administration. (b), The level of FPG, FINS, and HOMA‐IR in PCOS rats after administration for 4 weeks ($\bar{\chi }$ ± s, *n* = 10), FPG, fasting blood glucose; H‐Ber, high concentration of berberine; HOMA‐IR, homeostasis model assessment of insulin resistance; L‐Ber, low concentration of berberine; MH, metformin hydrochloride; PCOS, polycystic ovarian syndrome. ^▲^ *p* < 0.05, ^▲▲^ *p* < 0.01 vs. control group, ^★^ *p*<0.05, ^★★^ *p*<0.01 vs. PCOS group

As shown in Figure 1, the values of FPG, FINS, and HOMA‐IR of PCOS rats had no significant changes compared with the control group before administration. After 4 weeks of administration, compared with the control group, the levels of FPG, FINS, and HOMA‐IR were significantly increased in the PCOS rats. However, compared with the PCOS group, the level of HOMA‐IR in the PCOS+L‐Ber group was significantly decreased, and both the PCOS+H‐Ber and PCOS+MH groups showed significant decreases in their FPG, FINS, and HOMA‐IR levels. The results showed that PCOS rats treated with Ber were significantly more glucose tolerant compared to PCOS rats and were similar to those treated with MH.

#### The level of hormones in the serum of the rats

**Figure 2:**
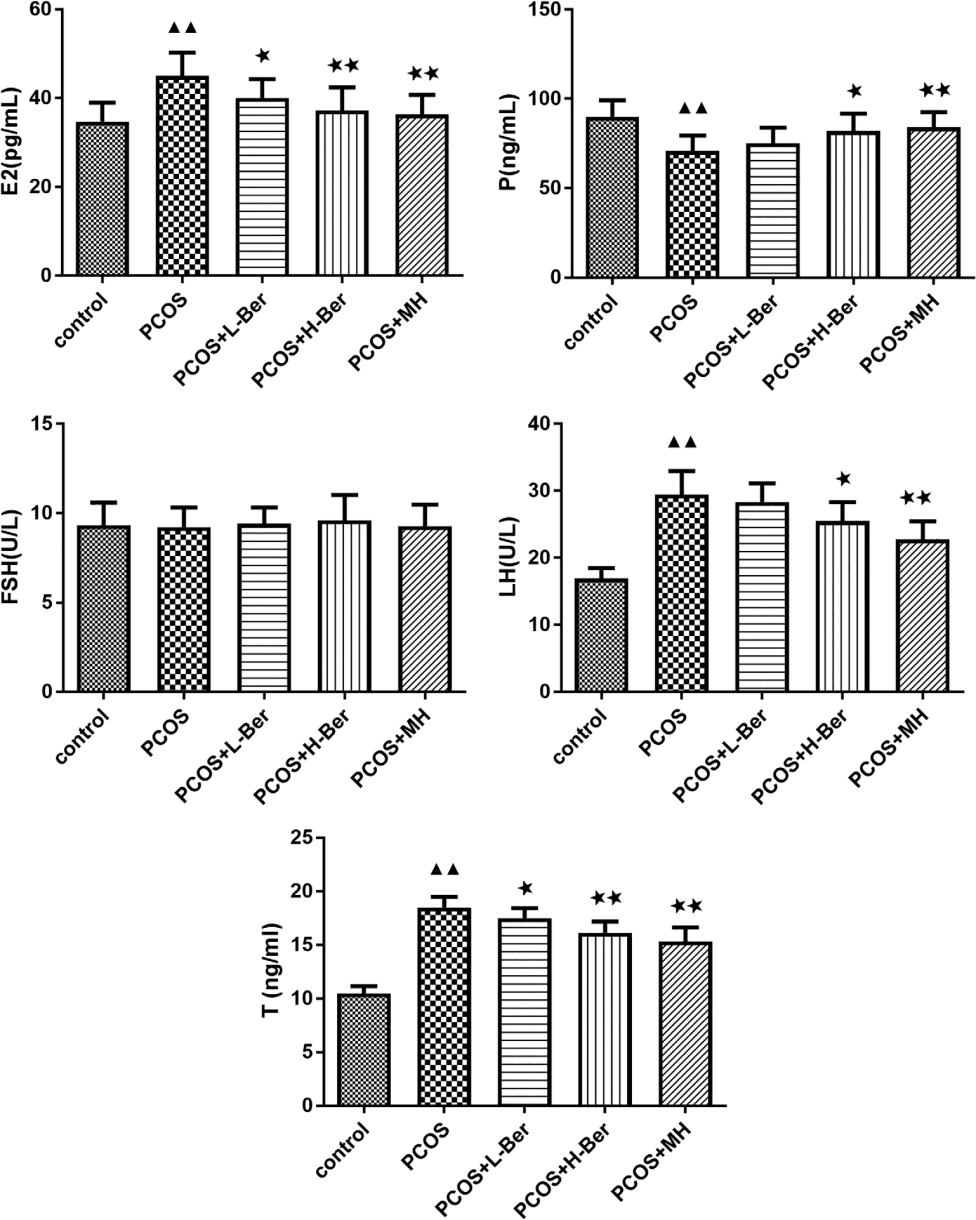
Comparison of hormone level detected by ELISA in the serum of rats ($\bar{\chi }$ ± s, *n* = 10), ELISA, enzyme‐linked immunosorbent assay; H‐Ber, high concentration of berberine; L‐Ber, low concentration of berberine; MH, metformin hydrochloride. ^▲^ *p* < 0.05, ^▲▲^ *p* < 0.01 vs. control group, ^★^ *p*<0.05, ^★★^ *p*<0.01 vs. PCOS group

Results of the ELISA assays indicated that the serum E2, T, and LH levels in the rats were significantly higher in the PCOS group than in the control group (*p* < 0.01), whereas the serum P level in the rats was significantly lower in the PCOS group than the control group (*p* < 0.01; Figure 2). At the same time, we observed the levels of E2 and T were significantly decreased (*p* < 0.01 and *p* < 0.05) while the levels of LH, FSH, and P had no significant change (*p* > 0.05) in the PCOS+L‐Ber group compared to the PCOS group. In addition, the level of P was increased and the levels of LH, E2, and T were suppressed in the PCOS+H‐Ber and PCOS+MH groups compared to the PCOS group. Furthermore, the level of FSH had no significant change across all groups (*p* > 0.05).

#### The influence of letrozole on the estrous cycle

**Figure 3:**
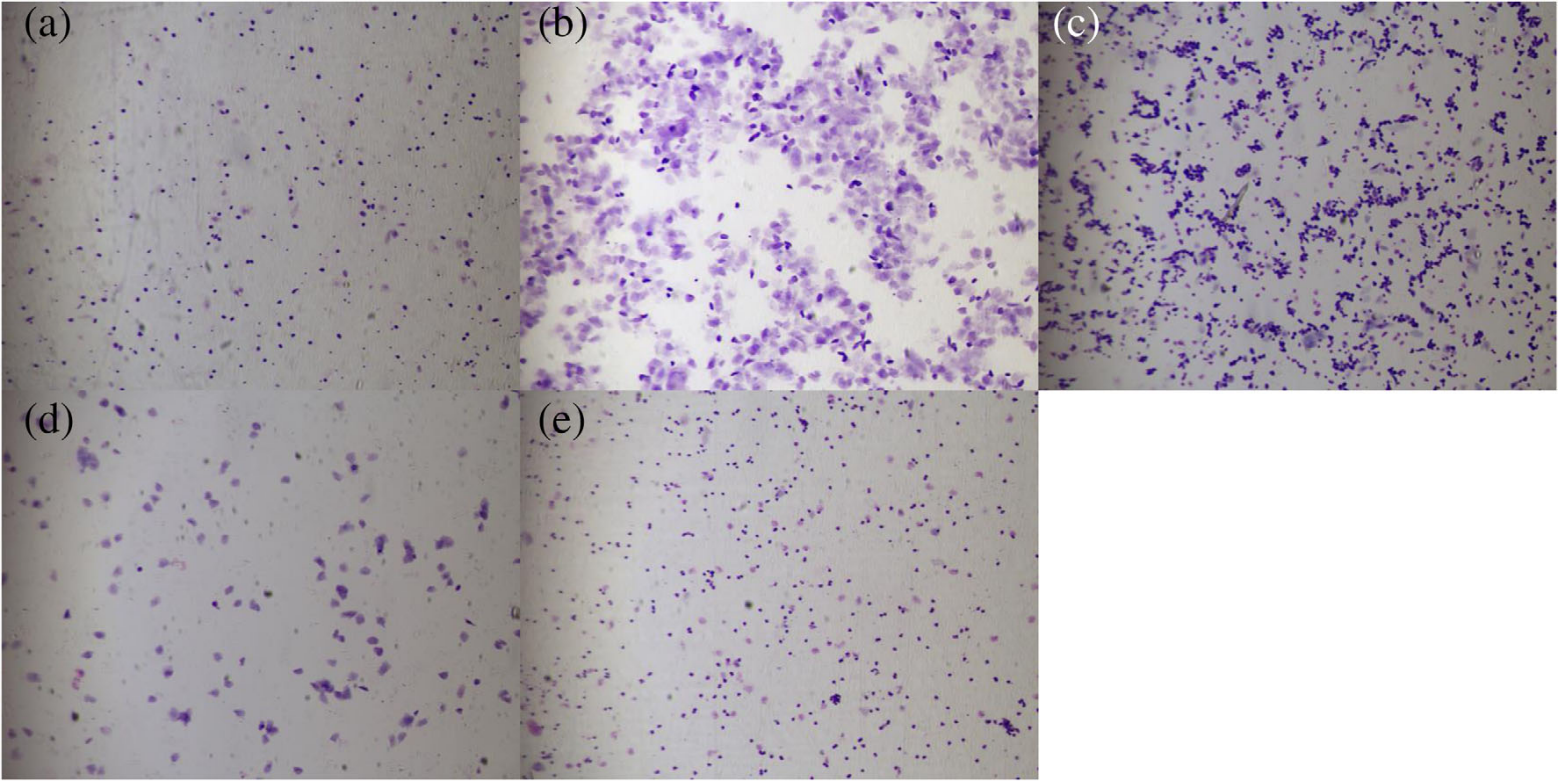
The vaginal smears of rats in the control and polycystic ovarian syndrome (PCOS). (a), The representative rat's vaginal smears from the control group in diestrus. (b), The representative rat's vaginal smears from the control group in estrus. (c), The representative rat's vaginal smears from the control group in metestrus. (d), The representative rat's vaginal smears from the control group in proestrus. (e), The representative rat's vaginal smears from the PCOS group predominantly exhibited leukocytes, the main cell type observed during the diestrus stage (original magnification, ×200)

Rats in the control group had 4–5 days estrous cycles, comprising proestrus, estrus, metestrus, and diestrus (Figure 3a‐d). The 10 rats in the PCOS group experienced prolonged diestrus after the letrozole gavage. Microscopy of stained smears of the vaginal secretions showed the presence of a large number of leukocytes, while few keratinized cells and epithelial cells were visible, suggesting anovulation. This suggested that the estrous cycle in the model group was irregular due to the gavage of letrozole.

#### Ovarian morphological changes

**Figure 4:**
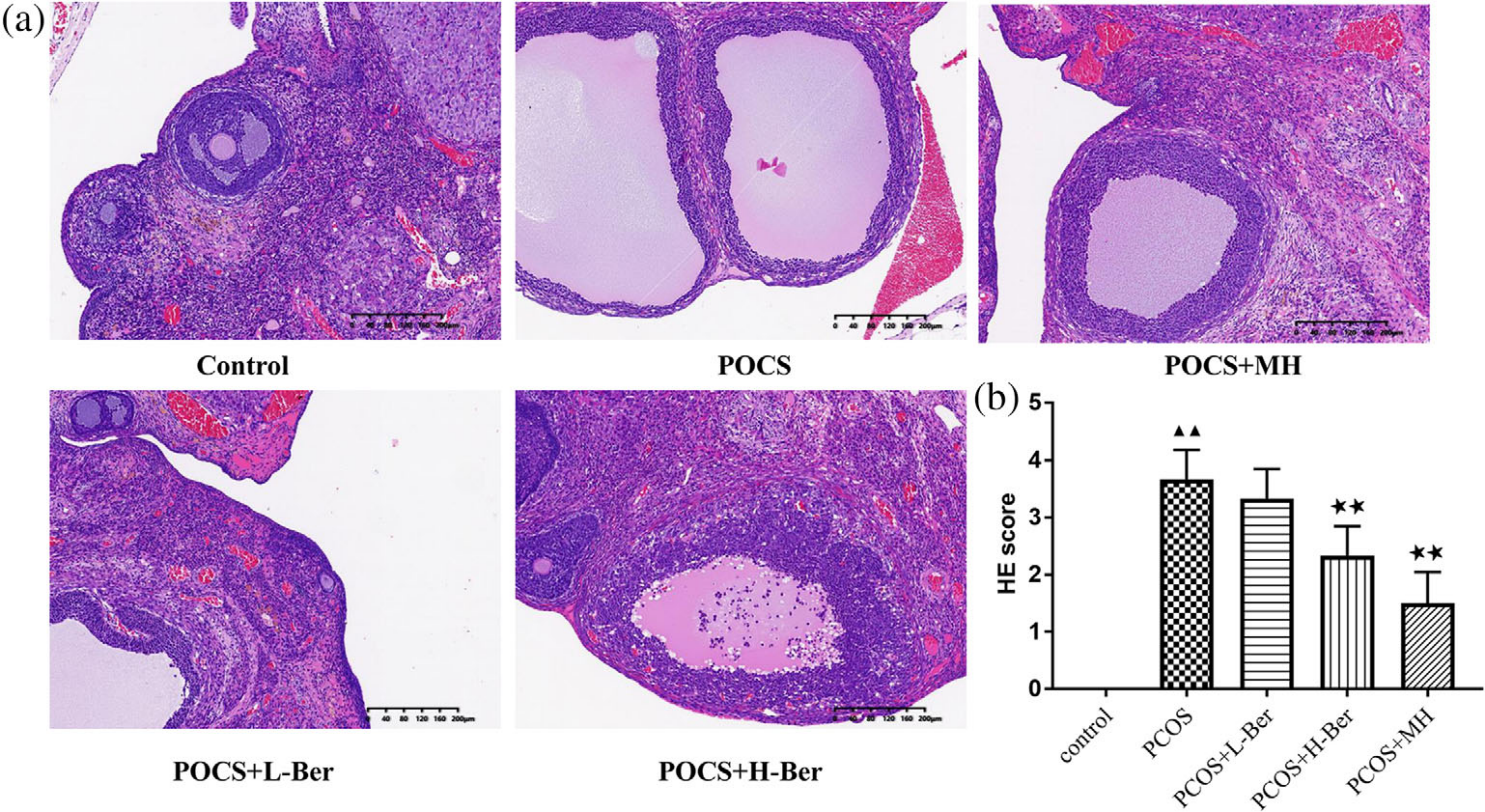
The morphological changes of the rats' ovarian tissues were evaluated by hematoxylin and eosin (H&E) staining. (a), representative microphotographs of H&E staining, original magnification × 100 ($\bar{\chi }$ ± s，*n* = 6); (b) Semiquantitative assessment of the histological lesions ($\bar{\chi }$ ± s，*n* = 6), H‐Ber, high concentration of berberine; L‐Ber, low concentration of berberine; MH, metformin hydrochloride; PCOS, polycystic ovarian syndrome. ^▲▲^ *p* < 0.01 vs. control group, ^★★^ *p*<0.01 vs. PCOS group

Light microscopy was applied to observe the rat ovarian structure as shown in Figure 4. Under light microscopy, the rat ovaries demonstrated multiple luteal, preantral, and antral follicles in the control group. The granular cells within the follicles showed multiple layers. Oocytes and corona radiata were visible in the follicles, part of which had been discharged. Compared with the control group, the PCOS group showed cystic dilatation follicle, lipid droplets in the cytoplasm, a small amounts of corpora lutea, and a thin layer of granulosa cells. Whereas in the administration group, it could be seen that the follicles were at different developmental stages, the corpora lutea was increased, the oocyte and corona were radiated in the mature follicle, and a thick layer of ordered granulosa cells with complete shapes were also observed.

#### Ber inhibits the apoptosis of ovarian tissue cells

**Figure 5:**
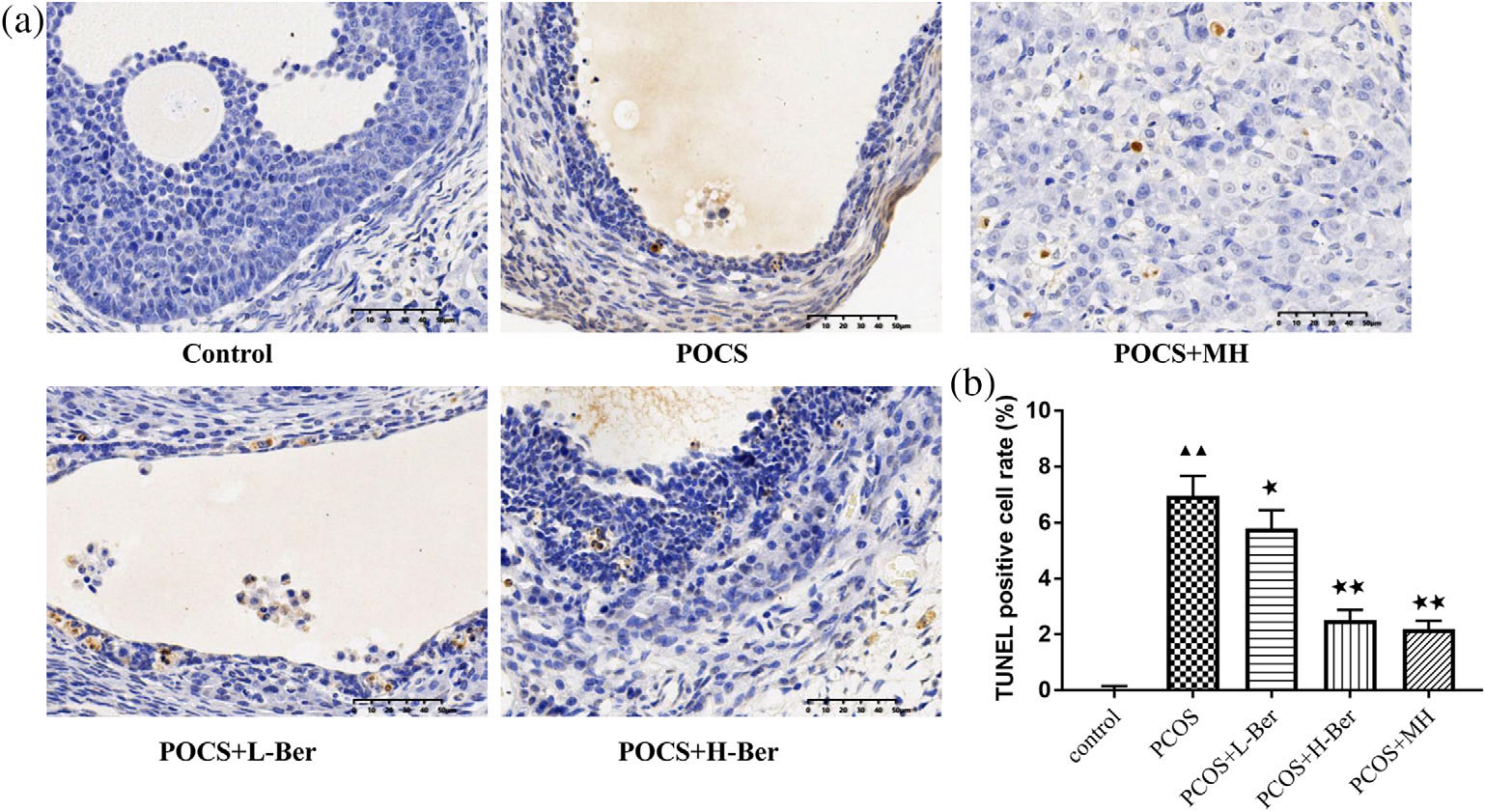
Effect of Ber and MH post‐treatment on the apoptosis of ovarian tissue cells. (a), Representative TUNEL staining images, original magnification ×400. (b) Semiquantitative assessment of the cell apoptosis, ($\bar{\chi }$ ± s，*n* = 3), H‐Ber, high concentration of berberine; L‐Ber, low concentration of berberine; MH, metformin hydrochloride; PCOS, polycystic ovarian syndrome. ^▲▲^ *p* < 0.01 vs. control group, ^★★^ *p*<0.01 vs. PCOS group

To investigate the apoptosis of ovarian tissue cells, we conducted TUNEL staining on the ovarian tissues and the results are shown in Figure 5. Compared with the control group, it demonstrated a higher number of apoptotic cells in the PCOS group. On the other hand, less TUNEL positive cells were observed in the ovarian tissue from rats after Ber treatment. At the same time, the number of TUNEL positive cells in the PCOS+MH group were also decreased significantly. Semiquantitative assessment confirmed this result showing that the number of TUNEL positive cells significantly increased immediately after the letrozole challenge, but decreased after treatment with Ber and MH.

### In vitro study

#### Ovarian granulosa cells identification

**Figure 6:**
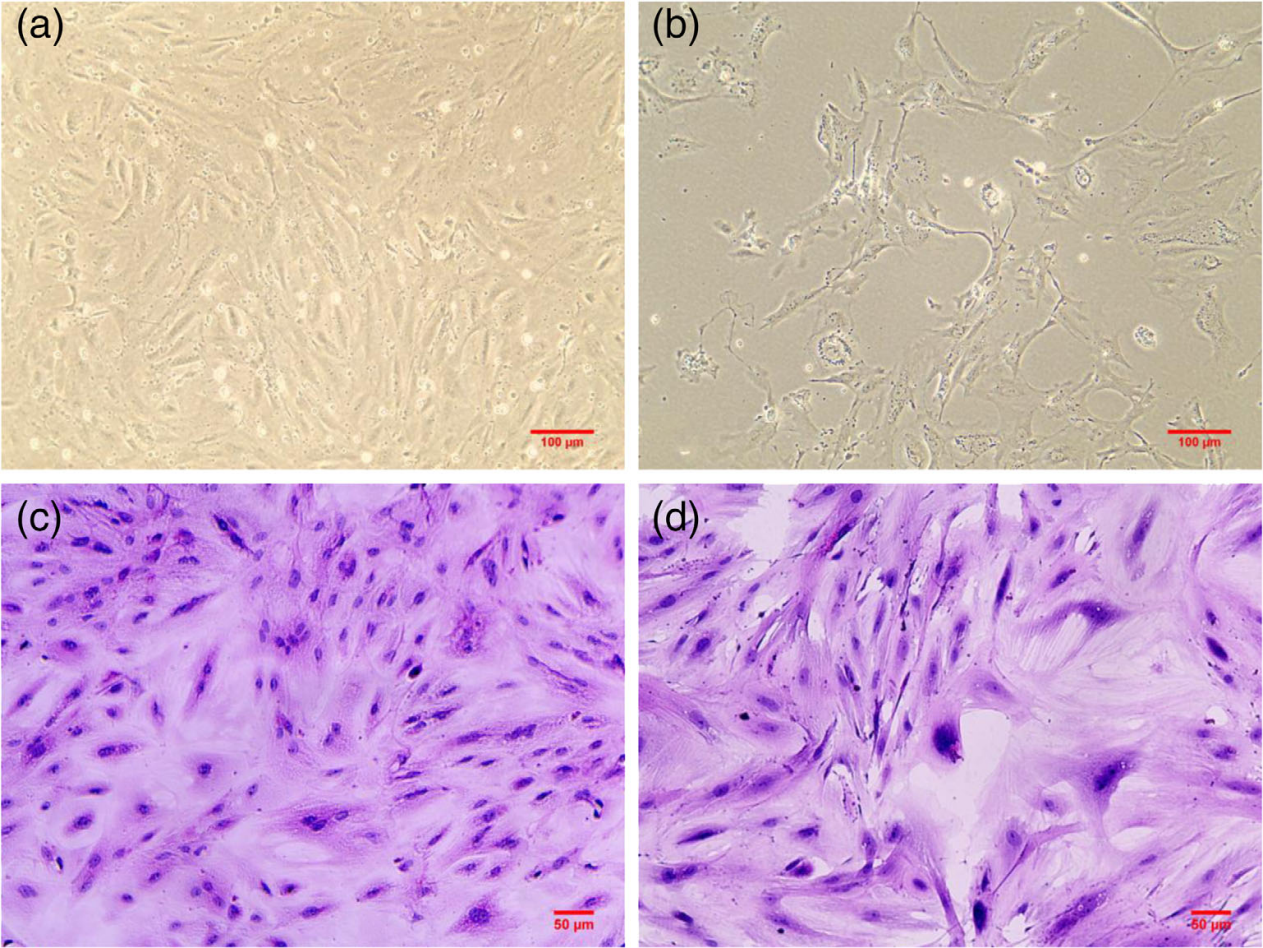
Identification of rat ovarian granulosa cells by hematoxylin and eosin (H&E) staining. (a) Morphology characteristic of ovarian granulosa cells in the control group (a) and the PCOS group (b) (original magnification, ×100). H&E staining images of ovarian granulosa cells in the control group (c) and PCOS group (d) (original magnification, ×200)

As shown in Figure 6, in the initial stage of ovarian granulosa cells developments, most of the cells were adherent and presented a rounded shape after 24 h of cell culture in the normal group, and the adherence time of the ovarian granulosa cells in the PCOS group was slightly later than that of the normal group. After 48 h of culture, the ovarian granulosa cells showed a pleomorphic or fusiform morphology in the normal group, while they showed pleomorphic or similar fibroblast morphology in the PCOS group. At the same time, the cells extended pseudopodia to connect with each other (Figure 6a, b). Then, we identified the ovarian granulosa cells by H&E staining and their morphological structure was observed under a light microscope. The nuclei of the normal ovaries were blue, and the cytoplasm was reddish and contained many particles (Figure 6c), but for the PCOS group, the nuclei were oval, and some nuclei were enlarged (Figure 6d) or polygonal.

#### Ber induces proliferative and anti‐apoptotic effects on granulosa cells

To investigate the effects of Ber exposure on ovarian granulosa cells in vitro, primary ovarian granulosa cells were exposed for various lengths of time at various concentrations. The effects of letrozole exposure on cell viability and growth were determined using a CCK‐8 assay at four exposure time points (12, 24, 48, and 72 h) with concentrations of 0.1 and 0.2 mg/mL. The results suggested that the cell viability was significantly reduced in the letrozole treated groups when compared with that in the control groups after culturing for the four exposure times. We found 0.2 mg/mL Ber treatment for 48 h significantly increased cell viability (*p* < 0.01). The 0.2 mg/mL dose of Ber was selected for subsequent experiments.

**Figure 7:**
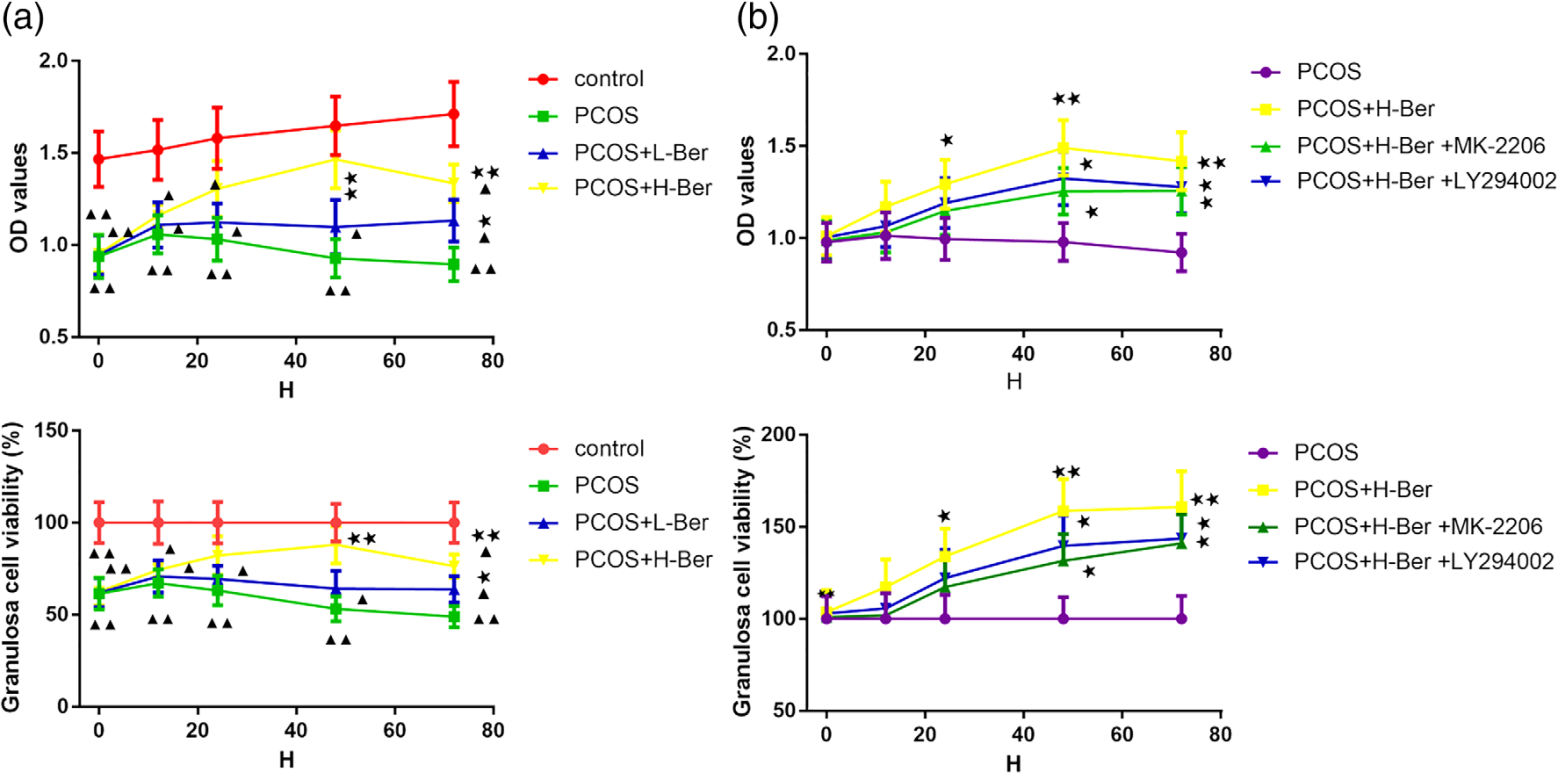
Effects of Ber treatment on the growth of ovarian granulosa cells in vitro. (a) Ovarian granulosa cells viability following treated with 0 (control), 0.1, or 0.2 mg/mL of Ber for 12, 24, 48, or 72 h. (b) Ovarian granulosa cells viability following treatment with the simultaneous addition of either LY294002 or MK‐2206 in combination with 0.2 mg/mL Ber, ($\bar{\chi }$ ± s，*n* = 3), H‐Ber, high concentration of berberine; L‐Ber, low concentration of berberine; PCOS, polycystic ovarian syndrome. ^▲^ *p* < 0.05, ^▲▲^ *p* < 0.01 vs. control group, ^★^ *p*<0.05, ^★★^ *p*<0.01 vs. PCOS group, ^#^ *p*<0.05, ^##^ *p*<0.01 vs. PCOS+H‐Ber group

**Figure 8:**
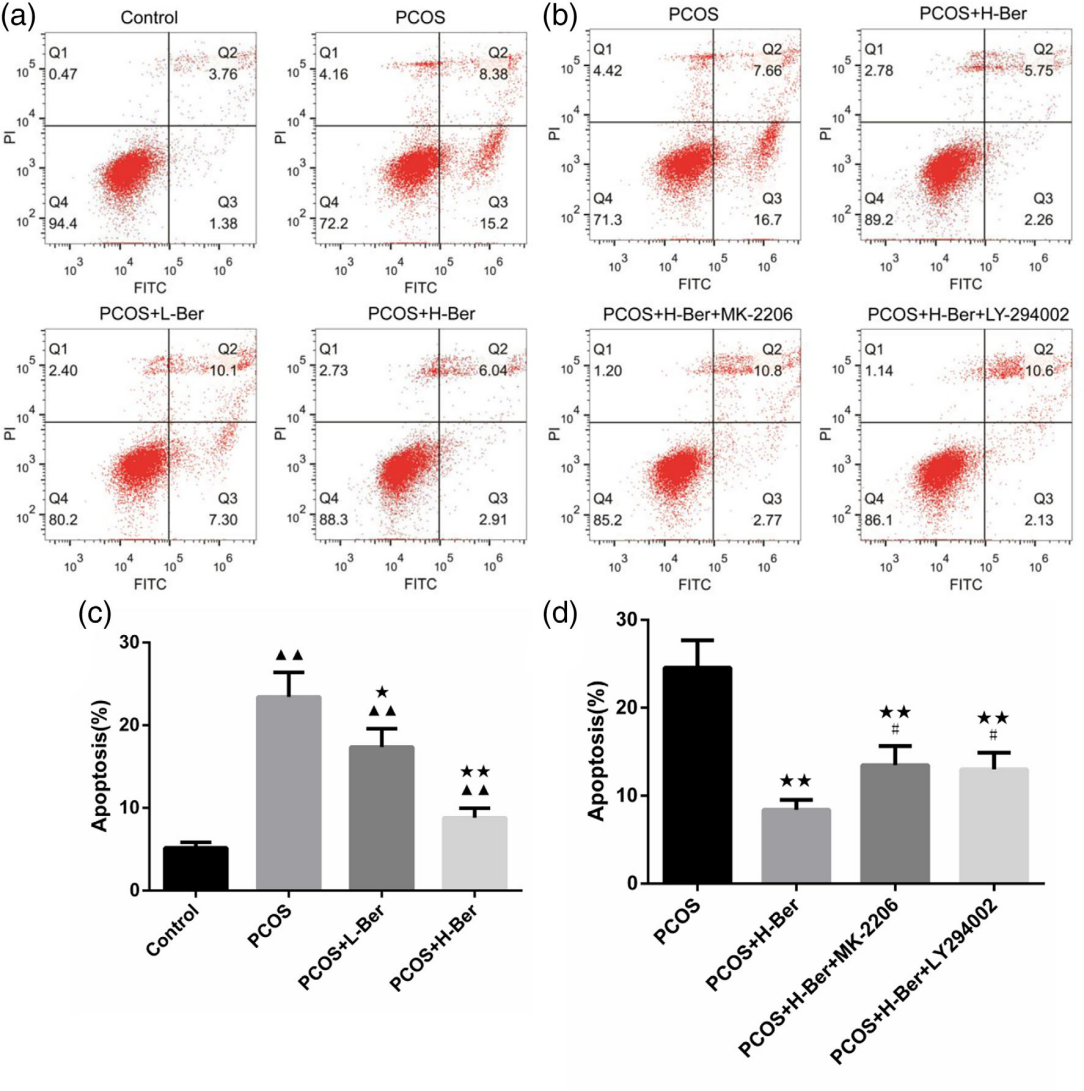
Effect of Ber on cell apoptosis after lead acetate induced injury in rat ovarian granulosa cells. (a and c), flow cytometry detection of apoptosis revealed that Ber significantly induced ovarian granulosa cell apoptosis. (b and ) flow cytometry detection of the anti‐apoptotic effects of Ber were blocked in combination with either 20 mM LY294002 or 20 mM MK‐2206. ($\bar{\chi }$ ± s，*n* = 3), H‐Ber, high concentration of berberine; L‐Ber: Low concentration of berberine; PCOS, polycystic ovarian syndrome. ^▲^ *p* < 0.05, ^▲▲^ *p* < 0.01 vs. control group, ^★^ *p*<0.05, ^★★^ *p*<0.01 vs. PCOS group, ^#^ *p*<0.05, ^##^ *p*<0.01 vs. PCOS+H‐Ber group

To further verify the involvement of the PI3K/AKT pathway in the stimulation of the growth of the ovarian granulosa cells, two pharmacological inhibitors, a PI3K inhibitor (LY294002, Beyotime Biotech) and an AKT inhibitor (MK‐2206, Sigma–Aldrich Co., LLC), were used. Cell proliferation and cell apoptosis were measured by the CCK‐8 assay and flow cytometry, respectively. In granulosa cells treated with 0.2 mg/mL Ber, a significant stimulatory effect of Ber on cell growth was observed (*p* < 0.05) compared with the PCOS group. The inhibitory effects of both 20 mM LY294002 and 20 mM MK‐2206 on cell growth were also confirmed. However, simultaneous addition of either LY294002 or MK‐2206 in combination with 0.2 mg/mL Ber blocked the letrozole‐induced cell growth but not significantly (*p* > 0.05). CCK‐8 analysis further revealed that 0.2 mg/mL Ber treatment significantly stimulated granulosa cell proliferation (*p* < 0.01), while this stimulatory effect was blocked in the presence of either LY294002 or MK‐2206 (*p* < 0.05) (Figure 7). In addition, flow cytometry analysis indicated that granulosa cell apoptosis was significantly inhibited by the addition of 0.2 mg/mL Ber (*p* < 0.01), but was elevated in the presence of either LY294002 or MK‐2206 (*p* < 0.05). Moreover, the anti‐apoptotic effects of Ber were blocked in combination with either LY294002 or MK‐2206 (Figure 8). These data confirmed that Ber exerts its proliferative and anti‐apoptotic effects on ovarian granulosa cells in a PI3K/AKT‐dependent manner.

#### Blockade of PI3K/AKT attenuates the modulatory effects of Ber on protein expression

**Figure 9:**
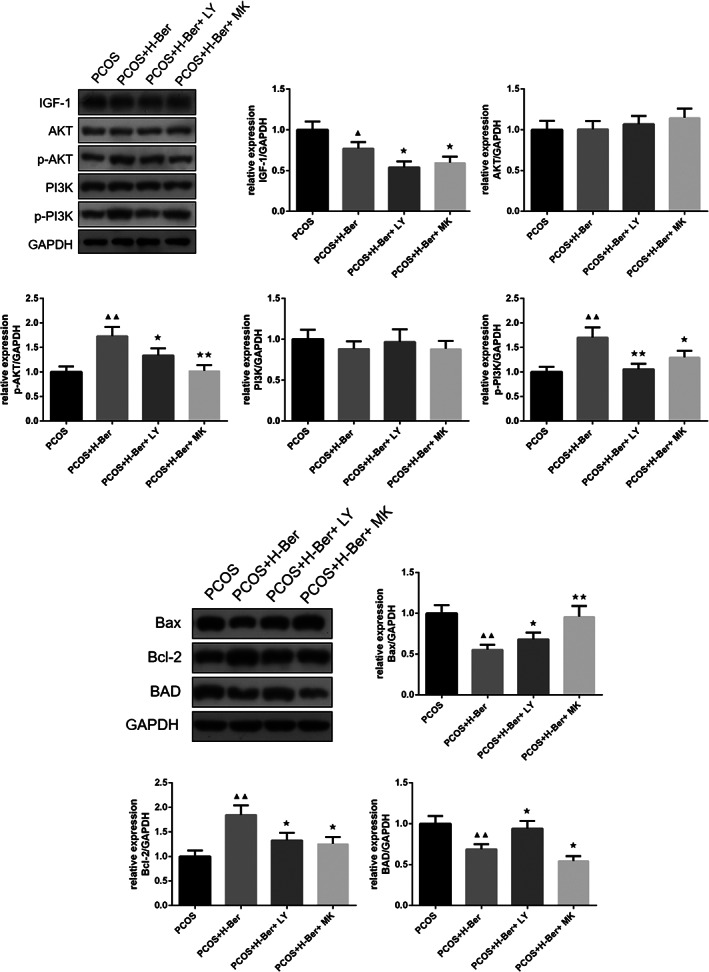
Effects of Ber in combination with either LY294002 or MK‐2206 on the protein expression of selected genes associated with cell apoptosis and regulation of the PI3K/Akt pathway. ($\bar{\chi }$ ± s，*n* = 3), H‐Ber, high concentration of berberine; PCOS, polycystic ovarian syndrome, L‐Ber: low concentration of berberine, ^▲^ *p* < 0.05, ^▲▲^ *p* < 0.01 vs. PCOS group, ^★^ *p*<0.05, ^★★^ *p*<0.01 vs. PCOS+H‐Ber group

As shown in the western blotting analysis, the expression level of AKT and PI3K was no significantly different in all groups, but the protein expression levels of Bcl‐2, p‐AKT, and p‐PI3K were significantly enhanced by 0.2 mg/mL Ber treatment (*p* < 0.01), while pharmacological inhibition of the PI3K/AKT pathway by LY294002 significantly decreased IGF‐1, Bcl‐2, p‐AKT, and p‐PI3K protein expression (*p* < 0.05 and *p* < 0.01) and MK‐2206 significantly decreased p‐PI3K, p‐AKT, and BAD protein expression (*p* < 0.05). In addition, the western blot assay also showed that LY294002 promoted the expression of BAD and Bax, while MK‐2206 promoted the expression of Bax in ovarian granulosa cells (Figure 9).

## Discussion

PCOS is defined as a metabolic syndrome.^19^, ^20^ The infertility that affects women with PCOS is thought to be partly attributable to IR, which affects up to 70% of those with PCOS and is linked to profoundly abnormal insulin activity in the reproductive system.^21^ Ber is an isoquinoline alkaloid compound that can be extracted from many different plants, and it has pharmacological activities that make it well‐suited to immune modulation, lowering glucose, and cholesterol levels, and combatting cancer or microbial pathogens.^22^, ^23^, ^24^ Multiple recent studies have explored the clinical use of Ber for treating PCOS.^25^

IR and the dysregulation of glucose metabolism are common in PCOS patients.^26^ Zhang et al^22^ found that the level of HOMA‐IR and the ovarian morphology in a PCOS model were restored after treatment with Ber. In this study, we elucidated the beneficial effects of Ber for PCOS through the establishment of a disease model in vivo and in vitro. A major characteristic of PCOS is an elevated serum androgen level.^27^, ^28^ In vivo experiment, we found that treated with Ber alleviated the serum hormone imbalance and improved HOMA‐IR. And the serums level of E2, LH, P, and T were decreased in PCOS treated with Ber and MH by ELISA assay. Meanwhile, this effect was comparable with that of the widely used medicine MH and thus shows great promise in the application of Ber for PCOS treatment.^23^, ^29^ Ber treatment could reduce the abnormal secretion of androgen and achieve a physiological androgen balance. In addition, by in vivo and in vitro experiments, we found that Ber positively regulated ovarian functions by promoting proliferation and inhibiting apoptosis of ovarian granulosa cells.

PI3K signaling cascades are important regulators of a host of cellular activities such as proliferation, differentiation, survival, apoptosis, and glucose homeostasis. Previous studies have shown that activation of the PI3K/AKT signaling pathway has important effects on insulin resistance.^30^ Long et al.^31^ created an insulin‐resistant in vivo model and confirmed that IR was related to the PI3K‐AKT signaling pathway. When insulin receptors are activated, they phosphorylate insulin receptor substrate (IRS), which then binds to the PI3K protein.^32^ The insulin signaling pathway is dependent upon signaling through a number of intermediaries, including IRS, PI3K, and AKT, thereby regulating cellular glucose intake.^33^ Zhang et al.^22^ found that berberine‐mediated effects against IR in a PCOS rat model were associated with its ability to enhance activation of PI3K/AKT signaling in these animals. In line with this result, our result indicated that the levels of FPG, FINS, and HOMA‐IR were significantly decreased after treatment with Ber.

The PI3K/AKT pathway may also be involved in the regulation of ovarian granulosa cell apoptosis and proliferation. Previous works suggested that granulosa cell apoptosis is involved in many ovarian physiological processes including the female germ cell cyst breakdown and primordial follicle assembly, follicular atresia, and luteolysis.^34^ In particular, ovarian granulosa cell apoptosis has been demonstrated to be an important mechanism for follicular atresia.^35^ Apoptotic proteins like Bcl‐2, Bax, and BAD were detected by western blot. The Bcl‐2 protein is anti‐apoptotic, but Bax and BAD are pro‐apoptotic. Both of them play key roles in the regulation of cellular apoptosis and exists a balance between pro and anti‐apoptotic components.^36^ The results showed high protein expression of pro‐apoptotic factors such as Bax and BAD and lower expression of Bcl‐2 in granulosa cells of the PCOS group after treatment with the simultaneous addition of either LY294002 or MK‐2206 in combination with 0.2 mg/mL Ber, with similar values to those found in the PCOS group. These results are consistent with the previous findings that PI3K and AKT inhibition have beneficial effects on PCOS.^37^ This confirmed that Ber inhibited the apoptosis of ovarian granulosa cells by the PI3K/AKT pathway.

## Conclusion

In summary, we reported on the beneficial effects of Ber in a PCOS rat model, including improvements in serum hormone levels, HOMA‐IR and the apoptosis and proliferation of ovarian granulosa cells. In addition, one of the important findings was the involvement of the PI3K/AKT pathway in the Ber‐induced beneficial effects on PCOS. This work provides experimental evidence to support the clinical application of Ber as a potential PCOS treatment.

## Author Contributions

Yu Jia designed the experiment. Wang Chenye and Hua Zhoujuan performed the experiment and acquisition the data. Yu Jia, Ding Caifei, Jiang Xuejuan and Hua Zhoujuan analyzed the data. Yu Jia and Wang Chenye obtained the funding and wrote the manuscript. Wang Chenye revised the manuscript. All authors discussed the results, and approved the final manuscript.

## Conflict of Interest

The authors declare that they have no conflict of interest.

## Data Availability

The data that support the findings of this study are available from the corresponding author upon reasonable request

## References

[1] Hadjiconstantinou M , Mani H , Patel N , Levy M , Davies M , Khunti K , et al. Understanding and supporting women with polycystic ovary syndrome: a qualitative study in an ethnically diverse UKsample. Endocr Connect. 2017;6:323–30. 28515051 10.1530/EC-17-0053 PMC5510451

[2] Curi DD , Fonseca AM , Marcondes JA , et al. Metformin versus lifestyle changes in treating women with polycystic ovary syndrome. Gynecol Endocrinol. 2012;28:182–5. 22309675 10.3109/09513590.2011.583957

[3] Busiah K , Colmenares A , Bidet M , Tubiana‐Rufi N , Levy‐Marchal C , Delcroix C , et al. High prevalence of polycystic ovary syndrome in type 1 diabetes mellitus adolescents: is there a difference depending on the NIH and Rotterdam criteria? Hormone Res Paediatr. 2017;87:333–41. 10.1159/000471805 28437788

[4] Ndefo UA , Eaton A , Green MR . Polycystic ovary syndrome: a review of treatment options with a focus on pharmacological approaches. P & T. 2013;38:336–8. 23946629 PMC3737989

[5] Rondanelli M , Infantino V , Riva A , Petrangolini G , Perna S . Polycystic ovary syndrome management: a review of the possible amazing role of berberine. Arch Gynecol Obstet. 2020;301:53–60. 32060683 10.1007/s00404-020-05450-4 PMC7028834

[6] Wu J , Li J , Li W , Sun B , Xie J , Cheng W , et al. Achyranthis bidentatae radix enhanced articular distribution and anti‐inflammatory effect of berberine in Sanmiao wan using an acute gouty arthritis rat model. J Ethnopharmacol. 2018;221:100–8. 29679725 10.1016/j.jep.2018.04.025

[7] Jin F , Xie T , Huang X , Zhao X . Berberine inhibits angiogenesis in glioblastoma xenografts by targeting the VEGFR2/ERK pathway. Pharm Biol. 2018;56:665–71. 31070539 10.1080/13880209.2018.1548627 PMC6319470

[8] Imenshahidi M , Hosseinzadeh H . Berberine and barberry (*Berberis vulgaris*): a clinical review. Phytother Res. 2019;33:504–23. 30637820 10.1002/ptr.6252

[9] Wei W , Zhao H , Wang A , Sui M , Liang K , Deng H , et al. A clinical study on the short‐term effect of berberine in comparison to metformin on the metabolic characteristics of women with polycystic ovary syndrome. Eur J Endocrinol. 2012;166:99–105. 22019891 10.1530/EJE-11-0616

[10] An Y , Sun Z , Zhang Y , Liu B , Guan Y , Lu M . The use of berberine for women with polycystic ovary syndrome undergoing IVF treatment. Clin Endocrinol (Oxf). 2014;80:425–31. 23869585 10.1111/cen.12294

[11] Schultze SM , Hemmings BA , Niessen M , Tschopp O . PI3K/AKT, MAPK and AMPK signalling: protein kinases in glucose homeostasis. Expert Rev Mol Med. 2012;14:e1. 22233681 10.1017/S1462399411002109

[12] Zheng J , Viacava FA , Kriwacki RW , Moldoveanu T . Discoveries and controversies in BCL‐2 proteins‐mediated apoptosis. FEBS J. 2016;283(14):2690–700. 26411300 10.1111/febs.13527

[13] Gwinn DM , Shackelford DB , Egan DF , Mihaylova MM , Mery A , Vasquez DS , et al. AMPK phosphorylation of raptor mediates a metabolic checkpoint. Mol Cell. 2008;30:214–26. 18439900 10.1016/j.molcel.2008.03.003 PMC2674027

[14] Tao R , Gong J , Luo X , Zang M , Guo W , Wen R , et al. AMPK exerts dual regulatory effects on the PI3K pathway. J Mol Signal. 2010;5:1. 20167101 10.1186/1750-2187-5-1 PMC2848036

[15] Sun J , Jin C , Wu H , Zhao J , Cui Y , Liu H , et al. Effects of electro‐acupuncture on ovarian p450arom, p450c17α and mrna expression induced by letrozole in pcos rats. Plos One. 2013;8:e79382. 24260211 10.1371/journal.pone.0079382 PMC3832614

[16] Zhu Q , Li YG . Berberine attenuates myocardial ischemia reperfusion injury by suppressing the activation of PI3K/AKT signaling. Exp Ther Med. 2016;11:978–84. 26998023 10.3892/etm.2016.3018 PMC4774358

[17] Hasegawa T , Kamada Y , Hosoya T , Fujita S , Nishiyama Y , Iwata N , et al. A regulatory role of androgen in ovarian steroidogenesis by rat granulosa cells. J Steroid Biochem Mol Biol. 2017;172:160–5. 28684382 10.1016/j.jsbmb.2017.07.002

[18] Shen H , Wang Y . Activation of tgf‐β1/smad3 signaling pathway inhibits the development of ovarian follicle in polycystic ovary syndrome by promoting apoptosis of granulosa cells. J Cell Physiol. 2018;234:11976–85. 30536903 10.1002/jcp.27854

[19] Azziz R , Carmina E , Chen Z , Dunaif A , Laven JSE , Legro RS , et al. Polycystic ovary syndrome. Nat Rev Dis Primers. 2016;2:16057. 27510637 10.1038/nrdp.2016.57

[20] Liu R , Huang H , Zhang J . Analysis of Bromocriptine in the treatment of polycystic ovary syndrome: a systematic review. Chinese J0 Mod Appl Pharm. 2019;36:1813–8.

[21] Moulana M . Immunophenotypic profile of leukocytes in hyperandrogenemic female rat an animal model of polycystic ovary syndrome. Life Sci. 2019;220:44–9. 30708097 10.1016/j.lfs.2019.01.048 PMC6392450

[22] Zhang N , Liu X , Zhuang L , Liu X , Fang J . Berberine decreases insulin resistance in a PCOS rats by improving GLUT4: dual regulation of the PI3K/Akt and MAPK pathways. Regul Toxicol and Pharmacol. 2019;110:104544. 10.1016/j.yrtph.2019.104544 31778716

[23] Pirillo A , Catapano AL . Berberine, a plant alkaloid with lipid‐ and glucoselowering properties: from in vitro evidence to clinical studies. Atherosclerosis. 2015;243:449–61. 26520899 10.1016/j.atherosclerosis.2015.09.032

[24] Abushouk AI , Salem AMA , Abdel‐Daim MM . *Berberis vulgaris* for cardiovascular disorders: a scoping literature review. Iran J Basic Med Sci. 2017;20:503–10. 28656085 10.22038/IJBMS.2017.8674 PMC5478778

[25] Luo R , Liao Z , Song Y , Yin H , Zhan S , Li G , et al. Berberine ameliorates oxidative stress‐induced apoptosis by modulating ER stress and autophagy in human nucleus pulposus cells. Life Sci. 2019;228:85–97. 31047897 10.1016/j.lfs.2019.04.064

[26] Li W , Li Q . Dysregulation of glucose metabolism even in Chinese PCOS women with normal glucose tolerance. Endocr J. 2012;59:765–70. 22673295 10.1507/endocrj.ej12-0049

[27] Zhao H , Zhou D , Chen Y , Liu D , Chu S , Zhang S . Beneficial effects of heqi san on rat model of polycystic ovary syndrome through the PI3K/Akt pathway. DARU. 2017;25:21–33. 29020999 10.1186/s40199-017-0188-7 PMC5637260

[28] Rajan RK , M SS, Balaji B . Soy isoflavones exert beneficial effects on letrozoleinduced rat polycystic ovary syndrome (PCOS) model through antiandrogenic mechanism. Pharm Biol 2017;55:242–51. 27927075 10.1080/13880209.2016.1258425 PMC6130471

[29] Penzias A , Bendikson K , Butts S , Coutifaris C , Falcone T , Fossum G , et al. Role of metformin for ovulation induction in infertile patients with polycystic ovary syndrome (PCOS): a guideline. Fertil Steril. 2017;108:426–41. 28865539 10.1016/j.fertnstert.2017.06.026

[30] Li T , Mo H , Chen W , Li L , Xiao Y , Zhang J , et al. Role of the PI3K‐Akt signaling pathway in the pathogenesis of polycystic ovary syndrome. Reprod Sci. 2017;24:646–55. 27613818 10.1177/1933719116667606

[31] Long M , Zhou J , Li D , Zheng L , Xu Z , Zhou S . Long‐term overexpression of neuropeptide Y in hypothalamic paraventricular nucleus contributes to adipose tissue insulin resistance partly via the Y5 receptor. PLoS One. 2015;10:e0126714. 25993471 10.1371/journal.pone.0126714 PMC4436377

[32] Li Y , Kuang H , Shen W , Wu X . Letrozole, berberine, or their combination for anovulatory infertility in women with polycystic ovary syndrome: study design of a double‐blind randomised controlled trial. BMJ Open. 2013;3:e003934. 10.1136/bmjopen-2013-003934 PMC3845065 24282248

[33] Jeon HJ , Yoon KY , Koh EJ , Choi J , Kim KJ , Choi HS , et al. Dieckol improve insulin sensitivity through the regulation of the PI3K pathway in C57BL/KsJdb/db mice. J Food Nutr Res. 2015;3:648–52.

[34] Hussein MR . Apoptosis in the ovary: molecular mechanisms. Hum Reprod Update. 2005;11:162–77. 15705959 10.1093/humupd/dmi001

[35] Zhang TY , Sun XF , Li L , Ma JM , Zhang RQ , Li N , et al. Ochratoxin a exposure impairs porcine granulosa cell growth via the pi3k/akt signaling pathway. J Agric Food Chem. 2019;67:2679–90. 30650308 10.1021/acs.jafc.8b06361

[36] Li Y , Ma H , Xue J . Effects of polygala on the expression of apoptosisrelated proteins in hippocampus nerve cells of diabetic rats. Chin J Anatomy. 2013;36:206–9.

[37] Shah KN , Patel SS . Phosphatidylinositide 3‐kinase inhibition: a new potential target for the treatment of polycystic ovarian syndrome. Pharm Biol. 2016;54:975–83. 26459667 10.3109/13880209.2015.1091482 PMC11133948

